# Evolution of Volatile Compounds and Spoilage Bacteria in Smoked Bacon during Refrigeration Using an E-Nose and GC-MS Combined with Partial Least Squares Regression

**DOI:** 10.3390/molecules23123286

**Published:** 2018-12-11

**Authors:** Xinfu Li, Jiancai Zhu, Cong Li, Hua Ye, Zhouping Wang, Xiang Wu, Baocai Xu

**Affiliations:** 1 School of Food Science and Technology, Jiangnan University, Wuxi 214122, China; lixinfu316@126.com (X.L.); zjc01@163.com (J.Z.); cobralc@126.com (C.L.); yehua_2004@163.com (H.Y.); wangzp@jiangnan.edu.cn (Z.W.); 2 State Key Laboratory of Meat Processing and Quality Control, Yurun Group, Nanjing 211806, China; wuxiang985@126.com; 3 School of Food Science and Biology Engineering, Hefei University of Technology, Hefei 230009, China

**Keywords:** smoked bacon, volatile compounds, SPME-GC-MS, electronic nose, biogenic amines, partial least squares regression

## Abstract

The changes in the concentration of volatile organic compounds (VOCs) and biogenic amines (BAs) in smoked bacon during 45-day refrigerated storage is investigated using solid-phase micro-extraction coupled with gas chromatography-mass spectrometry and high-performance liquid chromatography. In total, 56 VOCs and 6 BAs were identified and quantified. The possible pathways leading to their formation are analyzed and considered as the potential signs of microbial activity, especially by specific spoilage microorganisms (SSOs). *Leuconostoc* and *Lactobacillus*, which levels increased markedly with the extension of storage time, were recognized as SSOs. An electronic nose (e-nose) was employed to determine the changes in concentration of the odor components per sample present within half an hour. Partial least squares regression was then carried out to analyze the correlation between SSO growth, metabolite concentration, BA accumulation, and e-nose response. The results show that ten VOCs (ethanol, 2-furanmethanol, 1-hexanol, 1-propanol, phenol, 2-methoxyphenol, acetic acid, 3-ethyl-2-cyclopenten-1-one, furfural, and ethyl hexanoate) and three BAs (putrescine, cadaverine, and tyramine) can be associated with the growth of SSOs. Thus, they can be adopted as potential indicators to evaluate and monitor the quality of the bacon and develop appropriate detection methods. E-noses can used to recognize odors and diagnose quality of bacon.

## 1. Introduction

The spoilage of meat due to the growth of microorganisms and their associated metabolic processes, especially a group of bacteria called specific spoilage microorganisms (SSOs), is responsible for great economical losses in the food industry [1,2,3,4,5]. Numerous studies have reported that SSOs are associated with detrimental biochemical changes, e.g., production of volatile organic compounds (VOCs), accumulation of biogenic amines (BAs), etc. [1,2,3].

The growth and metabolism of SSOs in meat products during storage frequently leads to the production of off-flavors/odors, acidity, and gas [6,7,8]. Of course, certain volatile compounds in cooked meats contribute much to their acceptability [9]. However, the accumulation of VOCs (microbial metabolites) from SSOs, e.g., organic acids, alcohols, aldehydes, ketones, esters, sulfur compounds, and amines, determines the extent to which ‘sensory spoilage’ occurs and gradually causes a decline in quality characteristics of the meat [2,10,11,12]. At the same time, BAs can occur as a result of the accumulation of the products from SSOs, which also relate to food spoilage [5,13,14].

The changes occurring in the microbiota and biochemistry of the meat during storage have been identified as a potential means of revealing the meat’s quality or freshness [15,16,17]. For instance, butanoic acid and acetoin are related to the growth of lactic acid bacteria (LAB) and other spoilage microbial groups in packed beef [10]. Furthermore, the production of 3-methyl-1-butanol by SSOs (e.g., *Pseudomonas spp.* and *Carnobacterium spp.*) in meat stored under air and/or vacuum packaging is responsible for its off-odor [2]. In addition, Mansur et al. found that several microbial VOCs, such as acetic acid, butanoic acid, and 2-butanone, can be used as potential spoilage indicators [18]. The quantitative determination of BA profiles (mostly tyramine, putrescine, and cadaverine which are considered potentially toxic to humans) is an important method of ascertaining the quality and freshness of food [14,19]. Monitoring changes in the volatile fractions can also help in determining freshness and occurrence of microbial spoilage [6,8,20].

Volatile fractions can be analyzed using solid-phase microextraction (SPME) coupled with gas chromatography and mass spectrometry (SPME-GC-MS). The SPME technique, for example, has been successfully applied to analyze the VOCs in different foodstuffs [21]. BAs in meat and meat products may be detected using high-performance liquid chromatography (HPLC). However, although SPME-GC-MS and HPLC are effective ways of identifying and quantifying chemical compounds such as flavors and liquid substances individually, they cannot distinguish among these samples in overall profiles [22].

Electronic noses (e-noses) are a relatively recent innovation which use an array of chemical sensors that can detect and recognize odors and therefore constitute an advantageous tool for the rapid and accurate diagnosis of food quality [23,24]. Used with appropriate mathematical methods, an e-nose has the ability to distinguish aroma patterns and monitor changes in odors/flavors and the results can be used for classification and quality control [25,26,27]. Studies have already demonstrated the potential use of e-noses to predict the shelf-life of packaged beef [23], milk [28], and raw materials [24]. Siegmund and Pfannhauser used an e-nose and GC-MS to investigate the changes of the volatile fraction of—cooked chicken during chill storage and obtained highly correlated results [29]. Arnold and Senter confirmed that an e-nose can be used to differentiate the VOCs produced by bacteria isolated from processed poultry, different bacterial species (e.g., *Salmonella enteritidis*, *Escherichia coli*) have different compounds as area percentage (e.g., alcohols and 1-propanol) [30]. However, very little research has focused on predicting the shelf-life of smoked bacon.

As a multivariate statistical method, partial least squares regression (PLSR) may be the optimal way of elucidating the correlations between multiple variables by reducing the dimensions of the original data set without losing any information [31]. A plane figure can be used to explain the differences and similarities between samples, and the corresponding load maps can be interpreted to discover what effects are responsible for the similarities between samples [32]. Therefore, PLSR analysis is more accurate and has great potential in quality control applications aimed at identifying spoilage.

Bacon is produced from belly pork and demand for it has increased markedly in China in recent years. However, its high moisture and nutrient content and mild heat treatment during production make it highly susceptible to microbial spoilage when it is stored and, as a result, the acceptability of the quality/flavour decreases progressively [10,21,33].

It is well known that spoilage in meat is related to detrimental biochemical changes. To the best of our knowledge, only a limited number of studies have been carried out to study the changes. In this article, the VOCs and BAs potentially produced by spoilage-associated microorganisms over a 45-day period of storage will be monitored and described and e-nose will be used to classify groups of samples, the correlation and contribution of them will be discussed. The main aim of this work was to identify several key substances related to deterioration for evaluating and monitoring the quality of bacon during storage and even for exploiting rapid, convenient, and sensitive detection methods.

## 2. Results and Discussion

### 2.1. Results of the E-Nose Analysis

The e-nose is designed to detect and discriminate between complex odors according to the sensitivity ranges of the sensors. Properties such as shelf-life and freshness can thereby be investigated for any material that gives out volatile compounds [34,35].

**Figure 1:**
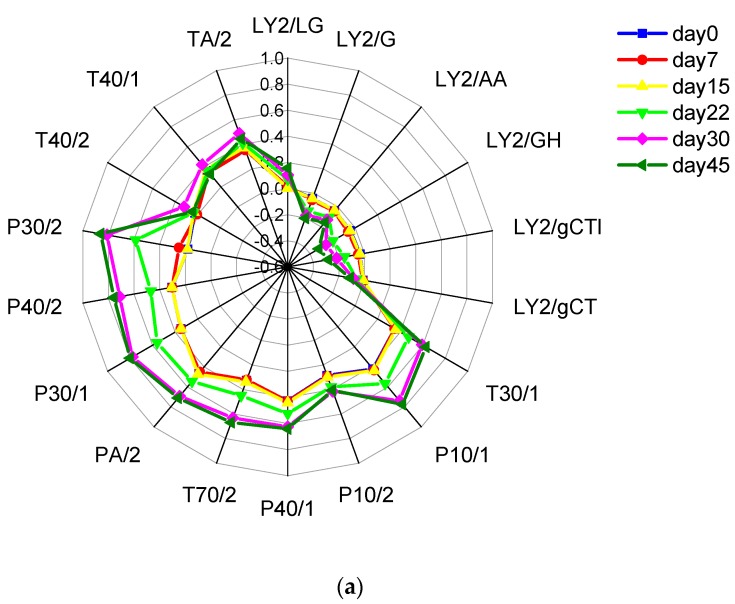

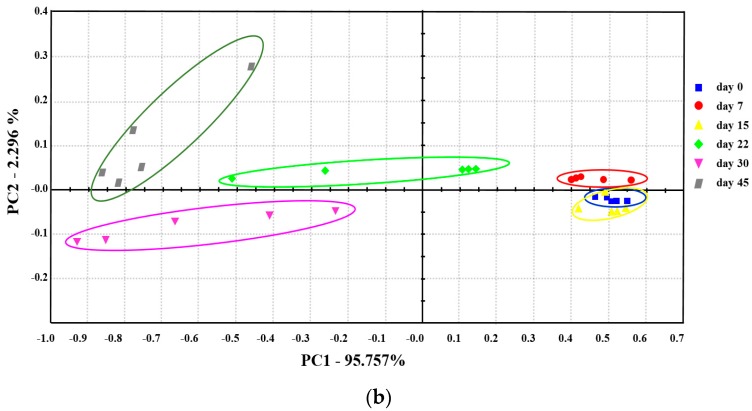
Results of radar plots and PCA of e-nose: (**a**) Radar plots of the mean responses of the sensors at different storage, each value represents the average score by five parallels with three replications. (**b**) Score plot using PCA analysis for discriminating bacon groups for different storage times at 4 °C. All 18 metal sensors were used as the inputs of PCA, PC1 and PC2 accounted for 95.7% and 2.3% of the variation, respectively. Each ellipse represents distances that are statistically equidistant from the group.

In this article, the e-nose was used to monitor changes in the bacon’s volatile compounds during storage. The mean sensor responses of the bacon are shown in Table S1. The radar chart shows how the average responses of the array of sensors differed with the samples (Figure 1a). The compounds that the sensors of the e-nose are sensitive to can therefore be investigated [36]. In general, the T- and P-type sensors gave responses that increased significantly (*p* < 0.05) with prolonged storage time, while the LY-sensors had responses that fell rapidly (*p* < 0.05). The response values may be used to infer which of the various volatile compounds are the principal ones resulting in the different responses of the e-nose and the different concentrations occurring during storage (the intensity of the corresponding sensor response increasing with increasing concentration).

In order to understand the e-nose identification process and the repeatability of the smoked bacon samples, the PCA method was used to analyze the multivariate data based on variable quantity restriction (Figure 1b, Table S2). In the figure, the representative data points for the bacon are plotted in a space whose axes are based on the first and second major components (principal components PC1 and PC2, respectively). The first two components account for 98.05% of the accumulative variance, which covers almost all the variable information. The data points from the samples are distinctly clustered on days 0, 7, and 15, indicating that the characteristic odors of these stored samples are very similar. However, the data points from day 22 are distributed within the first and second quadrants, a very different distribution to those of the previous samples. This indicates that the odor profile on day 22 was significantly different. Meanwhile, it is noteworthy that the points from day 30 are located in the third quadrant. Further change must have continued as the data points from day 45 were found to be distributed in the second quadrant. These results show that the e-nose performs well in identifying and differentiating between samples stored for over 15 days.

### 2.2. Microbiological Growth and Specific Spoilage Microorganisms

Microbiological data pertinent to the present analysis has been obtained in earlier research [37]. Briefly, the number of microorganisms increases sharply (by orders of magnitude) with prolonged storage. Culture-dependent experiments (traditional cultivation method) show that *Leuconostoc mesenteroides* and *L. carnosum* become the dominant species after 15 days. Culture-independent experiments (high-throughput sequencing (HTS)) indicate that LAB (*Leuconostoc* and *Lactobacillus*) prevail in the middle–late stages. These are considered to be SSOs. *Carnobacterium*, *Vibrio Brochothrix*, *Serratia*, *Fusobacterium*, *Rahnella*, and *Lactococcus* may also contribute to spoilage due to their proliferation or formation of metabolic products. The relative abundances of the top 10 species of spoilage bacteria at the genus level are shown in Table 1.

### 2.3. GC-MS Results for Bacon after Different Storage Times

For quantification purposes, a total of 56 calibration curves were drawn (Table 2). Among these compounds, phenols organic acids, alcohols and aldehydes, which had been reported as the most abundant compounds in Cantonese sausage [12], dry-cured ham [38], cold-smoked salmon [8], and fermented sausage [39,40,41]. According to Table 3, 56 volatile compounds were identified and quantified over the storage period, these compounds belong to eight classes: nine aldehydes, eight alcohols, 13 phenols, nine ketones, five alkanes, six terpenes, four organic acids, and two other compounds.

### 2.4. Volatile Compound Evolution during Storage

Although there were some small fluctuations, the amount of volatile compounds present tended to increase during storage (Table 3). The total amount fell to 644 mg/kg on day 7 having been 1100 mg/kg on day 0; it then increased to 3042 mg/kg on day 45. Table 3 shows that aldehydes and phenols were the major components in the initial stages of storage, followed by ketones. At the same time, alcohols, organic acids, alkanes, and others were minor components among the volatiles.

Considering the total occurrence of the compounds, the alcohol and phenol content increased dramatically during storage and clearly dominated the VOCs both in numbers and in amounts produced. At the same time, ketones, alkanes, organic acids, and terpenes exhibited minimal changes in their content. Only the aldehyde content appears to decrease as storage is prolonged.

Pearson correlation coefficients were used to reveal the correlations between the independent variables of storage time and VOC concentration. The results demonstrated that there were strong correlations between alcohols, phenols, and organic acids during refrigerated storage. More precisely, all 56 compounds were analyzed and a total of 23 were found to be significantly correlated (*p* < 0.05), as can be seen from Table 3. Of these 23 compounds, 21 showed positive correlation over the storage period, and the other two were negatively correlated. In particular, ethanol, 2-furan-methanol, 1-hexanol, and 1-propanol (*r* equal to 0.982, 0.982, 0.977, and 0.930, respectively) and phenol and 2-methoxyphenol (*r* equal to 0.956 and 0.919, respectively) show a perfect positive correlation, as the bivariate correlation (*r*) exceeds 0.900 [42]. The acetic acid (*r* = 0.964) and 3-ethyl-2-cyclopenten-1-one (*r* = 0.916) levels in organic acids and ketones had a similar tendency. In contrast, of the 9 aldehydes present only furfural (*r* = −0.900) was perfectly negatively correlated.

### 2.5. Compounds Formed Related to Microbial Activity

To gain a better understanding of how flavor is generated in the bacon, it is necessary to identify the sources of the volatile compounds. The production of VOCs in meat products is mainly a result of thermally-induced reactions and microbial activity. The former, for instance, include fatty acid oxidation and the Maillard reaction [43], both of which depend on the processing temperature and time (as well as other factors, such as, the addition of spices, smoking, the raw materials, and brining) [40,41,44,45]. Generally, the corruption of meat is mostly caused by the microbial populations of *Enterobacteriaceae*, *LAB*, *Pseudomonads*, *Brochothrix thermosphacta*, and some *Clostridia* [2].

Aldehydes are believed to make an important contribution to the flavor of smoked bacon, due to their high concentrations and low thresholds [46,47]. Linear aldehydes can be produced by the oxidation of lipid volatiles during processing (and degradation during storage) so that a wide range of flavors and odors can be formed [6,12]. Microorganisms associated with corruption, e.g., *Pseudomonas spp.*, *Carnobacterium spp.*, and *Enterobacteriaceae* are able to produce the highest number of aldehydes in the storage process [2].

In the samples used in this article, nine aldehydes (mainly the linear aldehydes including nonanal, hexanal, octanal, and furfural) were present in large amounts in the early stages and constituted the largest proportion of the VOCs measured. Straight-chain aldehydes (e.g., nonanal, hexanal, and heptanal) can be produced by *Carnobacterium spp.* and *Enterobacteriaceae* bacteria which are found in HTS. However, these compounds, with the exception of the phenylacetaldehyde, significantly decrease in abundance during storage (*p* < 0.05). The behavior of aldehydes association with spoilage bacteria is difficult to understand because they continued to fall in abundance—it may, however, be simply because these aldehydes were rapidly oxidized to acids and subsequently esterified with ethanol [12,48].

Alcohols are potentially produced by SSOs such as LAB (*Leuconostoc*, *Lactobacillus*), *Carnobacterium spp.*, *Pseudomonas spp.*, *Staphylococcus*, and *Kocuria* during the storage of meat [2,4,8]. Metabolic pathways, such as amino acid metabolism, lipid oxidation, methyl ketone reduction, and proteolytic activity are related to the alcohols generated [2,12,49]. As the quality of the bacon degraded with increasing storage time, nearly all of the nine alcohols increased significantly (*p* < 0.05) in abundance (e.g., see results for 1-hexanol in Table 3). The alcohols 3-methyl-1-butanol and 1-hexanol have been found inoculated with *Enterococcus spp.*, *Carnobacterium spp.*, *C. divergens*, and *R. aquatilis*. However, their low concentrations indicate that they did not play crucial roles in the spoilage process. The most abundant alcohols are ethanol, 2-furanmethanol, 1-propanol, and 2-butanol, which may be produced by LAB.

Phenols and methoxyphenols are compounds frequently encountered in wood smoke [45], and are formed by the pyrolysis of lignin [41]. In the smoked bacon studied in this article, eight phenols and five methoxyphenols were found (one predominant component is 2-methoxyphenol, which comes from softwood biomasses) which agrees with the results of other studies [41,50]. During vacuum storage, these compounds (especially 2-methoxyphenol) increased significantly in number (*p* < 0.05) and were positively correlated. This increase may also be related to growth of bacterial cells, such as LAB (especially *enterococci* and *lactococci*) which play a positive role in the production of phenol from benzaldehyde and phenylalanine [6,41].

Ketones are often considered to be secondary products formed during lipid oxidation, alkane degradation, and dehydrogenation of secondary alcohols by bacteria [2,8]. The existence of the ketones is mostly connected with the presence of *Staphylococcus*, *Pseudomonas spp.*, *Enterobacteriaceae,* and *Carnobacterium spp*. In our experiments, seven derivatives of 2-cyclopenten-1-one and 2 methyl ketones were found, most likely originating from the Maillard reaction from complex carbohydrates involved during pyrolysis [51], or from incomplete β-oxidation [52]. In this paper, the low ketone content and their slightly increasing amounts may indicate that they could be correlated with the *Carnobacterium spp.*, *Serratia*, and *Rahnella* revealed by HTS.

The volatile fatty acids in the meat may have arisen from the hydrolysis of triglycerides and phospholipids [53]. The main source of volatile fatty acids in meat storage is *Br. Thermosphacta*, *Carnobacterium spp.*, and LAB [2]. In this work, only five organic acids were detected during storage. The main ones are acetic and butanoic acids, which showed significant increases in abundance (*p* < 0.05) and were positively correlated with storage time. Acetic and butyric acids can be derived from the glucose catabolism of hetero-fermentative LAB, e.g., *Leuconostoc carnosum*, *Lactobacillus spp.*, and the microbial transformation of threonine, which is commonly associated with meat spoilage [6,54,55,56]. In this study, LAB (principally *Leuconostoc* and *Lactobacillus*) increased dramatically in the bacon during storage [37]. Similar results have been obtained for sliced cooked pork shoulder [44] and cold-smoked salmon [57] during refrigerated storage.

The alkanes present can be ignored as they contribute little to the flavor [58]. The terpenoids are expected to have originated from the addition of brine [41,44]. However, they are not expected to be major contributors to the flavor due to their low concentrations.

### 2.6. BA Evolution during Storage

Changes in histamine, putrescine, cadaverine, tyramine, 2-phenylethylamine, tryptamine, spermidine, and spermine in smoked bacon during storage were determined following the HPLC method. The BA content of the smoked bacon during the storage period is shown in Table 4. Neither tryptamine or phenylethylamine were found in any of the samples and remained absent throughout the entire storage period, a result that has been found before in Spanish meat products and chicken meat [59].

Spermidine and spermine, which were detected at concentrations that remained relatively stable, are amines that occur naturally in meat products [59,60]. Pearson correlation was used to determine the relationship between the independent variables of storage time and BA concentration. In general, the BAs (except spermidine) were found to be positively correlated with storage time, especially putrescine, cadaverine, and tyramine which increased significantly over the 45 days (*p* < 0.05). According to the literature, the accumulation of BAs in bacon appears to originate as a result of bacterial activity [59]. For example, *Enterobacteriaceae* (containing *Serratia liquefaciens* and *Hafnia alvei*), *Staphylococci*, *Enterococci*, *Pseudomonas spp.*, and certain *Lactobacilli*, are reported to play a key role in the generation of putrescine, cadaverine, and tyramine in meat products [14,19,61]. In addition, *Pediococcus* strains are often responsible for the presence of histamine [62]. Furthermore, LAB and *Enterobacteriaceae* have been associated with the production of tyramine and cadaverine, respectively, in fermented sausages [63]. Dietary polyamine and putrescine accumulation is mainly due to the activity of microorganisms, including *pseudomonads* and *Enterobacteriaceae* [60].

In the work reported here, LAB dominated the middle–late stages and caused the production of decarboxylases and this led to an increasing tyramine concentration during storage. *Enterobacteriaceae* increased significantly on days 30 and 45 which may be responsible for the large amounts of putrescine and cadaverine detected on these days. The histamine content remained stable which may be due to the absence of *Pediococcus* and *Pseudomonas aeruginosa.*

### 2.7. Relationships between Samples, VOCs, E-Nose Sensors, and Spoilage Bacteria

We now consider the possible correlations between the characteristics of the gas sensors, e-nose identification results, variations in VOCs and BAs, and the change in spoilage bacteria present. To do this, the mean values accumulated from the physicochemical indicators and microbial characteristics were processed using ANOVA-PLSR.

**Figure 2:**
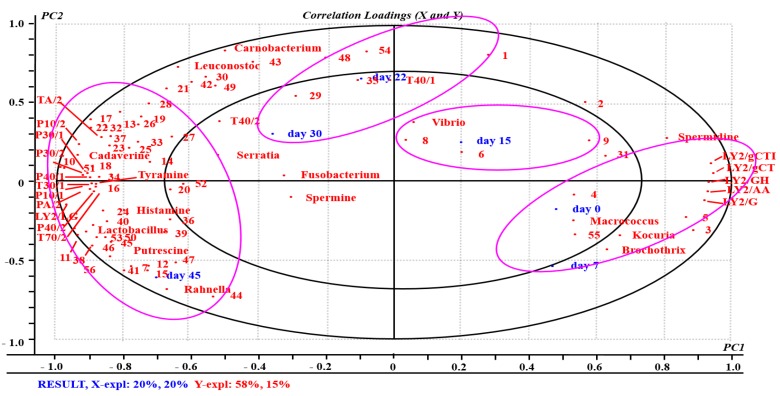
An overview of the variation found in the mean data from the partial least squares regression (PLSR) correlation loadings plot for bacon samples. The model was derived from e-nose sensors, VOCs, BAs and spoilage bacteria as the *X*-matrix and score values at different storage time as the *Y*-matrix. The concentric ellipses represent 100 and 50% explained variance, respectively. The code in PLSR corresponds to the 56 volatile compounds in Table 3. The 18 e-nose sensors are given in Figure 1a and Table S1. The regions within the purple ellipses are highly correlated with different variables.

The PLSR analysis involved using a total of 90 compounds as variables. The *X*-matrix was designated as the 18 e-nose sensors, 56 volatile compounds, six BAs, and 10 spoilage bacteria (Figure 2). The *Y*-matrix was generated using the variables scores after different storage periods. The output PLSR model consists of two vital PCs, which illustrate 81% of the variance of the cross-validation. Most of the variables are located between a small and a large ellipse (r^2^ = 0.5 and 1.0, respectively), which shows that the data is well described by the PLSR model [64].

As indicated in Figure 2, the samples can be divided into four groups, considering their locations, the resultant correlation between PC1 and PC2, and storage time. Firstly, the samples on days 0 and 7 lie in the positive region of PC1 and negative region of PC2. It is easy to see that the LY2 gas sensors (except LY2/LG), spermidine, *Brochothrix*, *Kocuria*, and *Macrococcus* are distributed in the same region surrounded mainly by aldehydes (furfural and 5-methyl-2-furancarboxaldehyde). Thus, these variables have good correspondence in the initial storage period before day 7. This phenomenon may be explained as follows. Most spoilage microorganisms are eliminated and inhibited by the mild heat treatment used during processing. The number of microbial counts did not differ significantly in the initial storage period showing that they could not be contributing to the formation of flavor substances. That is, the flavors and liquids present were mainly those naturally present in the meat product. Therefore, these results indicate that the original smoked bacon was of excellent quality.

Secondly, day 15 is located in the positive region of PC1 and PC2. It only seems to co-vary with aldehydes and *Vibrio*, and this is due to the situation being similar to that before day 7. These results are in accordance with the results of the PCA analysis of the e-nose data (see Figure 1b).

Thirdly, day 22 and day 30 lie in the negative region of PC1 and positive region PC2 surrounded by volatile compounds that are mainly alkanes, terpenes, and phenols, and correlated significantly with spoilage bacteria such as *Serratia* and *Fusobacterium.* The only BA nearby is spermine, and the data is well described by just two sensors: T40/1 and T40/2.

Fourthly, the samples on day 45 are in the negative region of PC1 and PC2. They co-varied with the responses of the T-type (excluding T40/1 and T40/2), P-type, and LY2-LG gas sensors, and are negatively correlated with the LY2 gas sensors (except LY2/G). These results indicate that the sensors have a great impact on differentiation between sample groups.

The results show that the e-nose can distinguish between and identify bacon in different stages of storage using the 18 sensors. Previous studies have shown that T- and P-type sensors are markedly related to sensory attributes such as off-flavors and odors [65,66]. Off-flavors/odors can be considered as being produced by SSO metabolites, are associated with lots of VOCs and usually contain aldehydes, alcohols, ketones, and others [2,67]. According to Figure 2, day 45 is significantly correlated with most of the volatile compounds (mainly alcohols, phenols, ketones and organic acids). As discussed previously in the article, they are spoilage-associated compounds produced by SSOs [2]. Combining this with the Pearson correlation analysis, the key VOCs are mainly 2-furan-methanol, ethanol, phenol, 2-methoxyphenol, and acetic acid. These compounds have higher concentrations and are significantly correlated (*p* < 0.05) with storage time. This shows that they make a greater contribution to flavor of the bacon as storage was prolonged.

Figure 2 also shows that day 45 co-varies with *Lactobacillus*, *Rahnella*, *Carnobacterium*, and *Leuconostoc.* These can thus be identified as the SSOs related to the spoilage of the bacon during storage. The current results are in good accord with those of Casaburi et al. [2] who proposed that *Enterobacteriaceae* and LAB are the SSOs associated with the spoilage of meat and production of many VOCs. Like other researchers, *Rahnella* [68], *Carnobacterium spp.* [69], and *Leuconostoc spp.* [70] have been found to play dominant roles (as SSOs) in the production of volatile compounds causing spoilage.

According to Figure 2, along the PC1 axis, BA accumulation mainly involves putrescine, histamine, tyramine, and cadaverine, which are located to the left of the plot and are positively correlated with the samples on day 45 and SSOs. BAs can be used as indicators of meat quality because they are metabolites derived from microbial growth and metabolism [59]. Therefore, the freshness of the bacon can be reflected by creating chemical quality and BA indices based on the measured levels of the BAs present (histamine, cadaverine, putrescine, and tyramine). As a result, early spoilage of the meat can be detected.

## 3. Materials and Methods

### 3.1. Sampling and Storage Conditions

Commercial samples of vacuum-packed smoked bacon were provided by Yurun (a joint venture between China and Italy whose factory is located in Ma’anshan in China). Bacon was produced from liquid smoke and sampled immediately after packaging. Samples were sampled along the processing line and vacuum packed individually, and transferred to the lab with drikold (dry ice). Then, the packages were thawed and refrigerated (at 0–4 °C). Five different batches of manufactured bacon were sampled and for each production batch eighteen parallel samples were selected randomly, a total of ninety samples of smoked bacon were tested in experiments. The vacuum-packaged bacon produced on that day was immediately analyzed (corresponding to ‘day zero’); the others after 7, 15, 22, 30, and 45 days of refrigerated storage (at 0–4 °C). On each sampling points, samples were withdrawn in triplicate for the subsequent biochemical analyses. The specifications of the manufacturer are as follows: sizes of polyethylene bag 110 × 220 mm, slice thickness 2.5 mm, 8–9 slices and 200 g per bag.

### 3.2. Electronic Nose

The e-nose employed (a Fox 4000, Alpha MOS, Toulouse, France) was furnished with an HS100 autosampler (Alpha MOS), 18 metal oxide sensors, and propriety data processing software (Alpha Soft v8.0) and was used to analyze the volatile compounds from the bacon. The 18 sensors specifically used are referred to as LY2/AA, LY2/G, LY2/gCT, LY2/gCTL, LY2/GH, LY2/LG, P10/1, P10/2, P30/1, P30/2, P40/1, P40/2, PA/2, T30/1, T40/2, T70/2, T40/1, and TA/2.

Minced samples (3.0 g) were placed in a 10 mL glass vial. The headspace generation were incubated at 50 °C for 10 min. Headspace gas (2000 μL) was pumped into the sensor chamber for 10 s at a constant rate of 150 mL min^−1^. The injection volume was 800 μL and the injected speed syringe was 2500 mL min^−1^. The sensor-response data were acquired for 120 s and the time between injections was set to 600 s.

On each sampling point (0, 7, 15, 22, 30, and 45 days), samples were withdrawn in triplicate for the sub-sequent analyses, each sample was analyzed in three times. A total of ninety samples were tested in experiments. Based the data of the triplicate and three replications, the average results were used for PCA analysis to obtain a stable result. The software was used to calculate the discrimination index—the higher the index, the better the difference [71].

### 3.3. Chemicals

Authentic standards were obtained from commercial sources: 3-methylbutanal, hexanal, furfural, heptanal, octanal, phenylacetaldehyde, α-pinene, 5-methyl-2-furancarboxaldehyde, nonanal, decanal, ethanol, 1-propanol, 2-butanol, 3-methyl-1-butanol, propylene glycol, 1-pentanol, 2-furanmethanol, 1-hexanol, phenol, 2-methylphenol, 4-methylphenol (*p*-cresol), 3-methylphenol, 2-methoxyphenol, 2,6-dimethylphenol, 2-methoxy-3-methylphenol, creosol, 4-ethyl-2-methoxyphenol, 2-methoxy-4-vinylphenol, 2,6-dimethoxyphenol, octane, eugenol, *trans*-isoeugenol, 1-hydroxy-2-propanone, 2-cyclopentenone, cyclooctane, 2-methyl-2-cyclopentenone, 3,4-dimethyl-2-cyclo-pentenone, benzoic acid, 2-hydroxy-3-methyl-2-cyclopentenone, 3-methyl-2-cyclopentenone, acetic acid, 2,3-dimethyl-2-cyclopentenone, 3-ethyl-2-cyclopentenone, butanoic acid, styrene, 3-ethyl-2-hydroxy-2-cyclopentenone, 2,3,4-trimethylpentane, 2-furylmethylketone 2,3,3-trimethylpentane, 2,2,8-trimethyldecane, propanoic acid, 3-methyl-3-heptene, (*Z*)-2-octene, d-limonene, and ethyl hexanoate were purchased from Sigma-Aldrich (Shanghai, China). 2-Octanol (internal standard) and *n*-alkane standards (C_6_–C_30_) were purchased from Sigma-Aldrich Chemical Co. (St. Louis, MO, USA). All substances are AR, at least 97% purity. The purification system from Milli-Q (Millipore, Bedford, MA, USA) to obtain purified water.

### 3.4. SPME-GC-MS of Volatile Organic Compounds in Bacon

A 4.0 g sample of minced bacon was put into a 20 mL glass vial. The bottle was sealed with a Teflon cover and placed in a water bath at 40 °C for 15 min. Extraction was performed using a previously-described method, with some slight modification [45]. A 65 μm polydimethylsiloxane/divinylbenzene SPME-fiber was exposed to the headspace of the sample (with stirring) at 40 °C for 40 min. It was then inserted into the thermal desorption system at 250 °C for 5 min.

GC-MS analysis was performed using a GC system (Agilent 7890) equipped with SPME and a mass-selective detector (MSD; type 5975, Agilent Technologies, Palo Alto, CA, USA). Samples were analyzed on DB-5 column (60 m × 0.25 mm × 0.25 μm; Agilent). The injection port was programmed to 250 °C, then retained for 5 min, and then the sample was injected in splitless mode. The carrier gas (helium; purity = 99.999%) flowed at a rate of 1.0 mL min^−1^. The MSD was used for chemical identification. The ionization energy voltage was set to 70 eV and the temperatures of the ion source, quadrupole mass filter, and transfer line to 230, 150, and 250 °C, respectively. The total ion current was monitored to record the chromatograms, and the scanning range was 40–450 *m*/*z*. The volatile compounds were identified according to the retention indices, retention times with those obtained for authentic standards, or with appropriate mass spectra libraries (Wiley, MD, USA, NIST, 2011). The C_6_–C_30_ saturated alkanes standard mixture was used for calculating retention indices (RIs). Each sample was subjected to GC-MS analysis in triplicate.

### 3.5. Calibration of Standard Curves

To get a matrix similar to bacon, model solution was prepared containing 30 mg/g hexadecanoic acid, 15 mg/g stearic acid, 30 mg/g oleic acid, 15 mg/g linoleic acid, 40 mg/g Glu, 40 mg/g Ala and 40 mg/g His in Milli-Q deionized water [52,72].

Seven levels of calibration: 1:5, 1:10, 1:20, 1:30, 1:40, 1:50 and 1:100 strengths were generated in triplicate. 2-Octanol (400 mg L^−1^ in water, 500 μL) was introduced into 4 g of model solution in a 20-mL vial and then extracted by SPME, using the same conditions as for bacon.

The calibration curves for determination of volatile compounds in extracts by GC-MS after SPME was established. Each point is the mean of six replicates. The standard curves were shown in the research (Table 2), where y represented the peak area ratio (peak area of volatile standard/ peak area of internal standard, A_x_/A_i_) and x represented the concentration ratio (concentration of volatile standard/concentration of internal standard, C_x_/C_i_). Fifty six volatile compounds were quantified to construct the standard curves [73].

### 3.6. Biogenic Amine Determination

Standard amine solutions were prepared according to previously developed procedures [74]. All the standard chemicals were obtained from Sigma Chemical Co. (St Louis, MO, USA) and CNW (Darmstadt, Germany). The extraction, purification, and separation steps were carried out according to the methods previously reported [14].

A Waters Alliance e2695 HPLC instrument fitted with a Waters UV-visible 2489 detector (Waters Co., Milford, MA, USA), and a Merck-Hitachi D-2500 chromato-integrator. The column used for separation was a Lichrospher 100RP-C18 (4.6 mm i.d. × 150 mm, 5 μm, Merck, Darmstadt, Germany). The BAs were detected using radiation of wavelength 254 nm. The concentrations of eight BAs (histamine, putrescine, cadaverine, tyramine, 2-phenylethylamine, tryptamine, spermidine, and spermine) were thus determined. All analyses were carried out in triplicate and the quantitative unit used was mg amine/kg.

### 3.7. Microbiological Analyses

The method of culture-dependent to the present analysis can been obtained in earlier research [37]. Briefly, eight culture media at different temperatures and atmosphere conditions were used to cultivate different microbial groups and/or species during the storage of bacon. Then colonies were isolated from selective culture media and identified by the bacterial 16S r RNA sequence analysis. Gene fragment was amplified with the universal primers 27F/1492R.

The method of culture-independent can also been obtained in the previous research [37]. Concisely, DNA extraction directly from bacon samples, pyrosequencing for 16S V3-V4 rRNA were conducted by using primer pairs 341F and 802R. Illumina sequencing was performed at Novogene Bioinformatics Technology Co., Ltd. (Beijing, China). Then the pyrosequence data was analysis.

### 3.8. Quality Assurance/Quality Control (QA/QC)

Both QA/QC are essential for the proper functioning of an analytical laboratory and the integrity of the data it produces. No interference was detected in the blanks, parallel and duplicates of the routine analytical procedures for bacon samples. The instrument was calibrated daily with calibration standard. The relative deviation between the concentration point of the calibration curve and the actual value was <20%, otherwise the calibration curve will be redrawn. The recoveries of the target analytes obtained from real samples based on internal standard were in the range of 80–115%, and the relative standard deviation (RSD) was <7%. All concentrations were normalized and not corrected by surrogate recoveries. Adding low concentration mixed standard reserve solution to blank sample. The limits of detection (LODs, S/N = 3) were 0.2–12.2 μg/kg, and the limits of quantification (LOQs, S/N = 10) were 2.5–35.3 μg/kg. The QA/QC of HPLC is performed in the same way as GC/MS. The average percent recoveries ranging from 70% to 110%. The LODs and the LOQs values were set as 0.05–0.5 mg/kg and 0.08–1.7 mg/kg, respectively.

### 3.9. Statistical Analysis

The data from the experiments was analyzed using analysis of variance (ANOVA) tests and Pearson’s correlation coefficient ‘*r*’ was employed to test the relationships among the different variables. Calculations were performed using the statistical package SPSS v20.0 (SPSS Inc., Chicago, IL, USA). Post-hoc multiple comparisons were determined by the Tukey’s test with the level of significance set at *p* < 0.05. PLSR models were adopted to study the correlation of the physicochemical and microbial characteristics and their contribution to the storage periods. The 18 sensors, 56 volatile compounds, 6 BAs, and 10 spoilage bacteria as the *X*-matrix and the variables scores at different storage periods as the *Y*-variable. PLSR analyses were performed using Unscrambler software (v9.7, CAMO, ASA, Oslo, Norway)—details of the procedures used are as described previously [65].

## 4. Conclusions

The shelf-life of meat and meat products is frequently assessed by performing sensory analyses and monitoring changes in microbial levels over time. However, there are some disadvantages to such an approach: analyses can be misleading, lengthy, expensive, and destructive. The measurement generated by the electronic nose can be associated to classification, determination of bacon evolution. It can potentially be commonly used by institutions, manufacturers and even consumers tasked with food quality control. It is also sensible to consider monitoring changes in some of the chemical metabolites produced during meat spoilage as a potential tool for assessing quality. A rapid analysis method/tool is needed to accurately quantify the chosen indicators used to predict the remaining shelf-life of food products. Therefore, we traced the evolution of VOCs, BAs, and associated microbial populations in smoked bacon during a period of refrigerated storage. A subsequent PLSR analysis showed which SSOs were closely related to the biochemical changes associated with spoilage. As a result, it was found that key roles are played by ethanol, 2-furanmethanol, 1-hexanol, 1-propanol, phenol, 2-methoxyphenol, acetic acid, 3-ethyl-2-cyclopenten-1-one, furfural, and ethyl hexanoate as well as putrescine, cadaverine, and tyramine. These key compounds can therefore be potentially used as the indicators for evaluating the quality of bacon and predicting its remaining storage time. They can also be used for developing rapid, convenient, and sensitive detection methods, such as gas-phase and liquid-phase biosensors for on-pack shelf-life determination. Therefore, before these biosensors are developed and applied in industry, it is necessary to study and analyze the metabolites and bacteria more deeply.

## Figures and Tables

**Table 1:** The relative abundances of the 10 top species of spoilage bacteria (at the genus level) in the bacon after 0, 7, 15, 22, 30, and 45 days of refrigerated storage [37].

Spoilage Bacteria	Storage Time/Days						Pearson’s Correlation Coefficients	
	Day 0	Day 7	Day 15	Day 22	Day 30	Day 45	*r*	*p*
*Leuconostoc*	0.91 ± 0.44 ^c^	1.74 ± 0.44 ^c^	26.72 ± 15.09 ^b^	53.73 ± 18.97 ^a^	40.26 ± 21.17 ^a,b^	27.49 ± 12.85 ^b^	0.616	0.193
*Lactobacillus*	2.62 ± 1.16 ^c^	3.76 ± 1.85 ^c^	6.25 ± 3.76 ^c^	9.45 ± 8.48 ^b,c^	18.70 ± 6.90 ^b^	41.94 ± 16.14 ^a^	0.934 **	0.006
*Vibrio*	5.91 ± 4.80 ^b^	5.42 ± 2.51 ^b^	30.69 ± 30.27 ^a^	10.96 ± 16.22 ^a,b^	8.08 ± 14.84 ^a,b^	8.44 ± 17.32 ^a,b^	−0.022	0.968
*Brochothrix*	2.43 ± 0.73 ^a,b^	12.76 ± 20.75 ^a^	1.48 ± 0.48 ^a,b^	2.41 ± 2.77 ^a,b^	0.17 ± 0.07 ^b^	0.33 ± 0.21 ^b^	−0.527	0.282
*Serratia*	0.34 ± 0.33	0.47 ± 0.34	0.20 ± 0.07	0.80 ± 1.19	6.28 ± 12.89	1.57 ± 2.83	0.48	0.335
*Kocuria*	13.19 ± 6.90 ^a^	4.48 ± 2.07 ^b^	1.56 ± 1.15 ^b^	0.33 ± 0.22 ^b^	0.38 ± 0.14 ^b^	0.36 ± 0.33 ^b^	−0.76	0.079
*Macrococcus*	8.58 ± 9.59 ^a^	0.97 ± 0.29 ^b^	0.27 ± 0.25 ^b^	0.01 ± 0.00 ^b^	0.15 ± 0.15 ^b^	0.01 ± 0.00 ^b^	−0.657	0.156
*Carnobacterium*	1.00 ± 0.37 ^c^	1.51 ± 0.47 ^c^	9.66 ± 6.62 ^a,b^	12.94 ± 8.14 ^a^	12.15 ± 6.38 ^a,b^	5.52 ± 1.29 ^b,c^	0.48	0.335
*Rahnella*	0.12 ± 0.07	0.48 ± 0.55	0.05 ± 0.03	0.13 ± 0.13	0.27 ± 0.07	2.78 ± 5.73	0.745	0.089
*Fusobacterium*	1.02 ± 1.18	0.89 ± 1.45	0.16 ± 0.20	0.09 ± 0.14	3.58 ± 0.36	0.91 ± 1.79	0.261	0.618
Others	63.88 ± 2.93 ^a^	67.51 ± 19.52 ^a^	22.98 ± 14.39 ^b^	9.15 ± 3.49 ^b^	9.97 ± 2.61 ^b^	10.65 ± 1.82 ^b^	−0.826 *	0.043

Figures in the table are means and standard error. ^a,b,c^ Means within a row refer to the significant difference at *p* < 0.05 according to Tukey’s multiple range test. Means in the same row with no superscript letters after them or with a common superscript letter following them are not significantly different (*p* < 0.05). * Significant at the 0.05 level; ** Significant at the 0.01 level.

**Table 2:** The standard curve, validation range and coefficient of determination (r^2^) for the volatile compounds in smoked bacon must. The equation based on the concentrations of peak areas and mean of six replicates at each of seven concentrations, total of 42 samples, “y” represents the peak area ratio and “x” indicates the concentration ratio. A model solution was used to test the quantities of the standards as described later.

No	Compound	Standard Curve	r^2^	Validation Range (μg kg^−1^)
1	3-Methylbutanal	y = 1.11x + 0.035	0.987	0.1–5
2	Hexanal	y = 1.84x − 0.019	0.991	10–100
3	Furfural	y = 0.94x + 0.083	0.986	10–100
4	Heptanal	y = 1.1x + 0.004	0.992	1–50
5	5-Methyl-2-furancarboxaldehyde	y = 0.88x + 0.031	0.982	1–50
6	Octanal	y = 0.73x − 0.041	0.984	1–100
7	Phenylacetaldehyde	y = 1.13x + 0.051	0.992	1–50
8	Nonanal	y = 2.12x + 0.006	0.979	20–500
9	Decanal	y = 0.95x − 0.081	0.981	1–50
10	Ethanol	y = 1.21x + 0.065	0.986	50–1000
11	1-Propanol	y = 0.95x − 0.027	0.988	50–1000
12	2-Butanol	y = 1.17x + 0.094	0.987	20–500
13	3-Methyl-1-Butanol	y = 1.18x + 0.028	0.992	1–10
14	Propylene glycol	y = 1.24x + 0.007	0.987	1–50
15	1-Pentanol	y = 0.81x + 0.057	0.981	1–50
16	2-Furanmethanol	y = 1.15x − 0.025	0.979	20–500
17	1-Hexanol	y = 2.28x + 0.068	0.973	1–50
18	Phenol	y = 1.51x + 0.029	0.986	10–100
19	2-Methylphenol	y = 0.86x + 0.021	0.992	10–100
20	4-Methylphenol (*p*-cresol)	y = 0.98x + 0.009	0.978	10–100
21	3-Methylphenol	y = 0.88x + 0.059	0.979	1–50
22	2-Methoxyphenol	y = 1.17x − 0.061	0.984	20–500
23	2,6-Dimethylphenol	y = 1.17x - 0.068	0.991	1–10
24	2-Methoxy-3-methylphenol	y = 0.88x + 0.069	0.992	1–10
25	Creosol	y = 0.84x + 0.029	0.994	20–500
26	4-Ethyl-2-methoxyphenol	y = 0.97x − 0.019	0.984	10–100
27	2-Methoxy-4-vinylphenol	y = 2.13x − 0.029	0.977	1–10
28	2,6-Dimethoxyphenol	y = 1.19x − 0.012	0.975	10–100
29	Eugenol	y = 1.16x + 0.068	0.987	1–10
30	*trans*-Isoeugenol	y = 0.96x + 0.043	0.984	1–10
31	1-Hydroxy-2-propanone	y = 1.15x + 0.042	0.986	1–50
32	2-Cyclopentenone	y = 0.93x − 0.051	0.989	1–10
33	2-Methyl-2-cyclopentenone	y = 0.82x − 0.067	0.978	10–100
34	3-Methyl-2-cyclopentenone	y = 1.19x + 0.018	0.983	10–100
35	3,4-Dimethyl-2-cyclopentenone	y = 0.99x + 0.085	0.984	0.1–5
36	2-Hydroxy-3-methyl-2-cyclopentenone	y = 0.96x − 0.027	0.991	10–100
37	2,3-Dimethyl-2-cyclopentenone	y = 0.85x + 0.047	0.978	10–100
38	3-Ethyl-2-cyclopentenone	y =1.17x + 0.018	0.979	1–50
39	3-Ethyl-2-hydroxy-2-cyclopentenone	y = 1.26x − 0.069	0.992	1–50
40	2,3,4-Trimethylpentane	y = 1.14x + 0.068	0.986	1–50
41	2,3,3-Trimethylpentane	y = 0.79x − 0.028	0.985	1–50
42	Octane	y = 0.86x + 0.021	0.985	1–50
43	Decane	y = 0.88x + 0.068	0.977	1–50
44	Cyclooctane	y = 1.18x − 0.012	0.978	1–50
45	2,2,8-Trimethyldecane	y = 0.096x + 0.035	0.986	1–50
46	3-Methyl-3-heptene	y = 0.99x − 0.039	0.982	1–10
47	(*Z*)-2-Octene	y = 0.86x + 0.017	0.981	1–10
48	Styrene	y = 0.94x − 0.008	0.994	1-10
49	α-Pinene	y = 1.27x + 0.058	0.989	0.1–5
50	D-Limonene	y = 1.17.x − 0.059	0.995	1–10
51	Acetic acid	y = 0.86x + 0.029	0.981	20–500
52	Butanoic acid	y = 0.84x − 0.046	0.993	1–50
53	Propanoic acid	y = 0.97x + 0.027	0.976	0.1–5
54	Benzoic acid	y = 0.86x + 0.038	0.977	1–50
55	2-Furylmethylketone	y = 0.98x − 0.017	0.988	1–10
56	Ethyl hexanoate	y = 1.18.x + 0.051	0.992	1–10

**Table 3:** Concentrations of VOCs (mg/kg) found for the bacon after 0, 7, 15, 22, 30, and 45 days of refrigerated storage, as measured using GC-MS.

Code ^A^	Compound	RI ^B^ (Calculated)	Identification ^C^	Storage Time/Days						Pearson’s Correlation Coefficients	
				Day 0	Day 7	Day 15	Day 22	Day 30	Day 45	*r*	*p*
	***Aldehydes***										
**1**	3-Methylbutanal	703	MS, RI, Sta	1.89 ± 0.35 ^a,D^	-	1.58 ± 0.55 ^b^	1.82 ± 0.35 ^a,b^	1.42 ± 0.35 ^c^	-	−0.373	0.467
**2**	Hexanal	803	MS, RI, Sta	97.61 ± 10.77 ^a^	38.98±4.89 ^c^	103.2 ± 6.02 ^a^	70.91 ± 7.86 ^b^	47.01 ± 10.08 ^c^	24.38 ± 3.19 ^d^	−0.644	0.167
**3**	Furfural	839	MS, RI, Sta	84.36 ± 7.13 ^a^	72.74 ± 4.03 ^b^	72.36 ± 8.49 ^b^	13.25 ± 2.05 ^c^	-	-	−0.900 **	0.007
**4**	Heptanal	904	MS, RI, Sta	33.7 ± 5.60 ^a^	0.71 ± 0.13 ^c^	8.22 ± 1.52 ^b^	-	3.4 ± 0.86 ^c^	-	−0.639	0.172
**5**	5-Methyl-2-Furan-carboxaldehyde	969	MS, RI, Sta	14.72 ± 2.26 ^b^	15.62 ± 1.54 ^a,b^	17.45 ± 0.90 ^a^	2.9 ± 0.35 ^c^	-	-	−0.844 *	0.035
**6**	Octanal	1006	MS, RI, Sta	88.70 ± 7.82 ^a^	24.89 ± 3.10 ^e^	79.15 ± 6.67 ^b^	53.53 ± 6.11 ^c^	40.92 ± 3.37 ^d^	53.8 ± 6.94 ^c^	−0.299	0.566
**7**	Phenyl-acetaldehyde	1052	MS, RI, Sta	1.49 ± 0.21 ^b^	-	-	2.88 ± 0.60 ^b^	1.87 ± 0.56 ^b^	36.98 ± 6.27 ^a^	0.779	0.068
**8**	Nonanal	1107	MS, RI, Sta	234.35 ± 28.98 ^b^	59.21 ± 7.11 ^e^	384.74 ± 27.84 ^a^	161.42 ± 20.22 ^d^	151.19 ± 16.09 ^d^	189.73 ± 19.80 ^c^	−0.069	0.897
**9**	Decanal	1209	MS, RI, Sta	13.72 ± 2.67 ^b^	3.94 ± 0.38 ^c^	17.65 ± 1.56 ^a^	2.54 ± 0.33 ^c^	2.08 ± 0.38 ^c^	-	−0.65	0.162
				570.54 ± 41.07 ^b^	216.09 ± 6.69 ^d^	684.35 ± 40.11 ^a^	309.25 ± 29.37 ^c^	247.87 ± 19.55	304.89 ± 23.41 ^c^	−0.421	0.406
	***Alcohols***										
**10**	Ethanol	<700	MS, RI, Sta	5.07 ± 0.85 ^d^	24.61 ± 2.72 ^d^	123.68 ± 12.65 ^c^	265.77 ± 39.00 ^b^	414.96 ± 52.38 ^a^	525.24 ± 78.14 ^a^	0.982 **	0
**11**	1-Propanol	<700	MS, RI, Sta	-	-	-	75.73 ± 10.402 ^c^	160.68 ± 20.27 ^b^	381.37 ± 48.94 ^a^	0.930 **	0.007
**12**	2-Butanol	<700	MS, RI, Sta	-	-	16.83 ± 2.93 ^b^	13.54 ± 3.66 ^b^	-	212.07 ± 32.92 ^a^	0.77	0.073
**13**	3-Methyl-1-butanol	735	MS, RI, Sta	-	-	6.06 ± 0.86 ^b,c^	5.07 ± 1.16 ^c^	10.44 ± 1.24 ^a^	7.56 ± 1.69 ^b^	0.819 *	0.046
**14**	Propylene Glycol	753	MS, Sta	5.47 ± 0.42 ^d^	1.94 ± 0.26 ^e^	12.66 ± 2.85 ^b^	11.51 ± 2.14 ^b,c^	6.81 ± 1.02 ^c,d^	17.32 ± 3.25 ^a^	0.74	0.093
**15**	1-Pentanol	767	MS, RI, Sta	0.59 ± 0.15 ^b^	-	-	2.58 ± 0.13 ^b^	-	37.43 ± 3.14 ^a^	0.764	0.077
**16**	2-Furanmethanol	861	MS, RI, Sta	27.39 ± 5.05 ^c^	28.39 ± 2.66 ^c^	63.5 ± 3.70 ^b^	66.29 ± 11.99 ^b^	66.83 ± 9.28 ^b^	113.76 ± 13.50 ^a^	0.982 **	0.003
**17**	1-Hexanol	871	MS, RI, Sta	1.37 ± 0.18 ^c^	1.14 ± 0.12 ^c^	15.41 ± 1.29 ^b^	38.12 ± 2.62 ^a^	37.23 ± 7.35 ^a^	41.24 ± 6.73 ^a^	0.905 **	0.013
				39.89 ± 4.73 ^d^	56.08 ± 1.62 ^d^	238.15 ± 16.41 ^d^	478.61 ± 41.06 ^c^	696.95 ± 61.40 ^b^	1335.99 ± 106.72 ^a^	0.976 **	0.001
	***Phenols***										
**18**	Phenol	990	MS, RI, Sta	10.33 ± 2.98 ^c^	12.4 ± 2.01 ^c^	18.62 ± 2.97 ^c^	28.51 ± 4.10 ^b^	49.11 ± 9.57 ^a^	52.62 ± 6.54 ^a^	0.956 **	0.003
**19**	2-Methylphenol	1064	MS, RI, Sta	16.41 ± 2.04 ^b^	19.1 ± 1.48 ^b^	37.76 ± 4.63 ^a^	41.73 ± 6.39 ^a^	33.58 ± 5.52 ^a^	41.74 ± 8.47 ^a^	0.796	0.058
**20**	4-Methylphenol (*p*-cresol)	1086	MS, RI, Sta	41.52 ± 4.48 ^c^	34.74 ± 3.78 ^c^	70.97 ± 7.45^. a,b^	45.12 ± 5.20 ^c^	54.16 ± 12.28 ^b^	76.45 ± 7.96 ^a^	0.706	0.117
**21**	3-Methylphenol	1088	MS, RI, Sta	2.11 ± 0.37 ^d^	-	12.1 ± 1.42 ^c^	45.13±4.26 ^a^	22.99 ± 3.49 ^b^	26.09 ± 4.39 ^b^	0.644	0.168
**22**	2-Methoxyphenol	1092	MS, RI, Sta	134.97 ± 16.79 ^d^	118.61 ± 11.19 ^d^	280.97 ± 26.87 ^c^	293.44 ± 21.90 ^b,c^	331.51 ± 44.66 ^a,b^	372.9 ± 47.34 ^a^	0.919 **	0.01
**23**	2,6-Dimethyl-phenol	1114	MS, RI, Sta	-	-	-	-	-	7.23 ± 0.83	0.758	0.081
**24**	2-Methoxy-3-methylphenol	1191	MS, Sta	1.16 ± 0.25 ^d^	1.34 ± 0.16 ^d^	4.98 ± 0.75 ^b^	3.49 ± 0.32 ^b,c^	3.1 ± 0.83 ^c^	8.19 ± 0.99 ^a^	0.850 *	0.032
**25**	Creosol	1194	MS, RI, Sta	49.2 ± 6.94 ^d^	34.45 ± 3.68 ^d^	95.11 ± 18.12 ^b,c^	108.1 ± 9.56 ^b^	69.6 ± 9.31 ^c^	123.38 ± 18.34 ^a^	0.768	0.074
**26**	4-Ethyl-2-methoxyphenol	1282	MS, RI, Sta	18.29 ± 2.57 ^d^	13.72 ± 1.98 ^d^	35.96 ± 3.68 ^b,c^	41.85 ± 6.86 ^a,b^	29.86 ± 3.16 ^c^	42.23 ± 9.39 ^a^	0.759	0.08
**27**	2-Methoxy-4-vinylphenol	1319	MS, RI, Sta	5.16 ± 1.09 ^c^	2.17 ± 0.44 ^d^	7.92 ± 1.24 ^a^	8.03 ± 1.51 ^a,b^	5.47 ± 0.77 ^b,c^	8.66 ± 1.46 ^a^	0.613	0.196
**28**	2,6-Dimethoxy-phenol	1356	MS, Sta	26.45 ± 2.63 ^b^	16.67 ± 1.81 ^b^	52.52 ± 8.82 ^a^	54.18 ± 8.64 ^a^	42.58 ± 6.82 ^a^	53.58 ± 9.49 ^a^	0.699	0.122
**29**	Eugenol	1362	MS, RI, Sta	1.25 ± 0.15 ^d^	0.29 ± 0.06 ^d^	2.55 ± 0.30 ^c^	12.66 ± 1.53 ^a^	-	4.4 ± 0.63 ^b^	0.251	0.631
**30**	*trans*-Isoeugenol	1458	MS, RI, Sta	1.27 ± 0.15 ^c^	-	3.48 ± 0.73 ^a^	3.05 ± 0.47 ^a,b^	2.98 ± 0.89 ^a,b^	2.51 ± 0.37 ^b^	0.539	0.27
				308.12 ± 20.85 ^c^	253.48 ± 8.10 ^c^	622.94 ± 37.94 ^b^	685.31 ± 22.89 ^b^	644.94 ± 79.80 ^b^	819.97 ± 87.33 ^a^	0.894 *	0.016
	***Ketones***										
**31**	1-Hydroxy-2-propanone	669	MS, RI, Sta	17.23 ± 2.23 ^b^	12.6 ± 2.64 ^b,c^	41.36 ± 6.70 ^a^	9.21 ± 0.94 ^c^	-	-	−0.548	0.26
**32**	2-Cyclopentenone	839	MS, RI, Sta	-	-	-	8.46 ± 1.41	7.9 ± 0.96	8.55 ± 1.84	0.847 *	0.033
**33**	2-Methyl-2-cyclo-pentenone	910	MS, RI, Sta	17.3 ± 2.41 ^c^	15.59 ± 1.62 ^c^	42.62 ± 6.15 ^b^	43.09 ± 6.86 ^b^	38.02 ± 4.25 ^b^	58.92 ± 5.99 ^a^	0.895 *	0.016
**34**	3-Methyl-2-cyclo-pentenone	971	MS, RI, Sta	19.03 ± 2.31 ^c^	20.6 ± 2.30 ^b,c^	34.75 ± 4.76 ^a^	25.02 ± 4.11 ^b^	36.99 ± 8.94 ^a^	40.73 ± 3.54 ^a^	0.867 *	0.025
**35**	3,4-Dimethyl-2-cyclopentenone	1027	MS, Sta	-	-	-	3.47 ± 0.75	-	-	0.065	0.902
**36**	2-Hydroxy-3-methyl-2-cyclo-pentenone	1034	MS, RI, Sta	15.23 ± 2.22 ^c^	3.66 ± 0.51 ^d^	40.12 ± 5.12 ^b^	-	36.85 ± 9.58 ^b^	61.47 ± 7.86 ^a^	0.708	0.115
**37**	2,3-Dimethyl-2-cyclopentenone	1042	MS, RI, Sta	17.43 ± 1.69 ^c^	16.12 ± 1.65 ^c^	40.2 ± 6.53 ^b^	43.6 ± 7.56 ^b^	33.79 ± 4.66 ^b^	52.96 ± 6.07 ^a^	0.855 *	0.03
**38**	3-Ethyl-2-cyclopentenone	1080	MS, Sta	3 ± 0.32 ^c^	3.43 ± 0.30 ^c^	6.53 ± 1.47 ^b,c^	6.12 ± 0.98 ^b,c^	7.52 ± 1.32 ^b^	17.91 ± 2.63 ^a^	0.916 *	0.01
**39**	3-Ethyl-2-hydroxy-2-cyclo-pentenone	1100	MS, RI, Sta	12.25 ± 1.59 ^c^	8.03 ± 2.62 ^d^	17.45 ± 2.04 ^b^	11.3 ± 1.64 ^c^	12.49 ± 1.83 ^c^	26.83 ± 4.06 ^a^	0.732	0.098
				101.47 ± 3.70 ^e^	80.01 ± 7.17 ^f^	223.03 ± 22.43 ^b^	146.8 ± 11.78 ^d^	173.56 ± 25.92 ^c^	267.38 ± 15.01 ^a^	0.807	0.052
	***Alkanes***										
**40**	2,3,4-Trimethyl-Pentane	752	MS, Sta	-	-	3.11 ± 0.62 ^c^	6.81 ± 1.77 ^b^	1.42 ± 0.30 ^c^	15.85 ± 2.77 ^a^	0.833 *	0.04
**41**	2,3,3-Trimethyl-pentane	759	MS, Sta	-	-	-	-	3.37 ± 0.69	33.35 ± 4.28	0.801	0.055
**42**	Octane	801	MS, RI, Sta	2.54 ± 0.33 ^c^	-	17.39 ± 2.94 ^b^	37.18 ± 5.44 ^a^	16.16 ± 2.54 ^b^	20.49 ± 3.47 ^b^	0.572	0.236
**43**	Decane	1000	MS, RI, Sta	19.72 ± 1.99 ^c^	3.48 ± 0.48 ^d^	13.4 ± 2.24 ^c^	72.29 ± 7.58 ^a^	48.09 ± 7.18 ^b^	22.49 ± 3.45 ^c^	0.343	0.506
**44**	Cyclooctane	1075	MS, Sta	1.24 ± 0.23 ^c^	3.12 ± 0.41 ^b^	4.41 ± 1.46 ^b^	-	-	15.02 ± 1.82 ^a^	0.631	0.179
				23.5 ± 1.94 ^e^	6.6 ± 0.74 ^f^	38.31 ± 1.32 ^d^	116.28 ± 11.83 ^a^	69.03 ± 6.12 ^c^	107.2 ± 5.38 ^b^	0.701	0.12
	***Terpene compounds***										
**45**	2,2,8-Trimethyl-Decane	792	MS, Sta	-	-	5.04 ± 1.23 ^b^	0.99 ± 0.53 ^c^	4.28 ± 0.49 ^b^	18.03 ± 3.03 ^a^	0.850 *	0.032
**46**	3-Methyl-3-heptene	798	MS, Sta	-	-	-	-	3.62 ± 0.75	8.13 ± 1.17	0.855 *	0.019
**47**	(*Z*)-2-Octene	807	MS, RI, Sta	-	-	-	-	-	5.15 ± 0.79	0.758	0.081
**48**	Styrene	898	MS, RI, Sta	-	-	-	10.44 ± 1.65	3.57 ± 0.34	-	0.172	0.745
**49**	α-Pinene	940	MS, RI, Sta	-	-	0.91 ± 0.23 ^b^	0.92 ± 0.14 ^b^	1.95 ± 0.38 ^a^	0.55 ± 0.12 ^c^	0.523	0.287
**50**	D-Limonene	1036	MS, RI, Sta	-	0.88 ± 0.17 ^c^	-	-	5.74 ± 0.83 ^b^	8 ± 1.42 ^a^	0.867 *	0.025
				-	0.88 ± 0.17 ^e^	5.95 ± 1.09 ^d^	15.82 ± 2.09 ^c^	19.16 ± 0.60 ^b^	39.87 ± 3.97 ^a^	0.975 **	0.001
	**Organic acids**										
**51**	Acetic acid	<700	MS, RI, Sta	24.78 ± 3.14 ^e^	10.72 ± 2.71 ^f^	44.88 ± 6.66 ^d^	64.73 ± 9.02 ^c^	100.71 ± 11.31 ^b^	126.69 ± 16.48 ^a^	0.964 **	0.002
**52**	Butanoic acid	890	MS, RI, Sta	9.89 ± 0.85 ^c^	2.62 ± 0.50 ^d^	9.36 ± 1.87 ^c^	3.68 ± 0.92 ^d^	22.57 ± 2.97 ^a^	16.22 ± 1.10 ^b^	0.6	0.208
**53**	Propanoic acid	985	MS, RI, Sta	-	-	-	-	0.64 ± 0.17	1.10 ± 0.19	0.890 *	0.017
**54**	Benzoic acid	1549	MS, RI, Sta	12.26 ± 2.56 ^b,c^	1.79 ± 0.20 ^e^	15.22 ± 3.47 ^a.b^	16.71 ± 4.48 ^a^	9.73 ± 1.98 ^c,d^	8.26 ± 1.74 ^d^	0.008	0.989
				46.93 ± 4.70 ^f^	19.39 ± 2.87 ^e^	69.46 ± 7.13 ^d^	85.12 ± 7.60 ^c^	133.65 ± 13.43 ^b^	152.27 ± 14.41 ^a^	0.935 **	0.006
	**Others**										
**55**	2-Furylmethyl-ketone	914	MS, RI, Sta	-	11.26 ± 1.21	9.11 ± 1.15	-	-	-	−0.437	0.386
**56**	Ethyl hexanoate	998	MS, RI, Sta	-	-	-	2.97 ± 0.90 ^c^	5.05 ± 1.09 ^b^	14.28 ± 1.67 ^a^	0.920 **	0.009
				-	11.26 ± 1.21 ^b^	9.11 ± 1.15 ^c^	2.97 ± 0.90 ^e^	5.05 ± 1.09 ^d^	14.28 ± 1.67 ^a^	0.523	0.287
				1090.45 ± 47.93 ^d^	643.79 ± 13.25 ^e^	1891.29 ± 127.58b ^c^	1840.16 ± 51.26 ^c^	1990.21 ± 207.91 ^b^	3041.85 ± 140.65 ^a^	0.918 **	0.01

^A^ Code representing the 56 volatile compounds used in the PlSR analysis. ^B^ The retention index of volatile compounds on DB-5 columns. ^C^ Method of identification: MS, mass spectrum comparison using Wiley library; RI, retention index in agreement with literature value; Sta, confirmed by authentic standards. ^D^ Figures in the table are means and standard error. The letters ^a–f^ refer to the significant difference at *p* < 0.05 according to Tukey’s multiple range test. Means in the same row with no superscript letters after them or with a common superscript letter following them are not significantly different (*p* < 0.05). - Not detected in sample. The letter “r” represents the relationship value between the variables of different storage days and the concentrations of VOCs, −1 to 0 is negative correlation, 0 no correlation, and 0 to +1 positive correlation. The letter “*p*” refer to the value of the significant difference. * Significant at the 0.05 level; ** Significant at the 0.01 level.

**Table 4:** BA concentrations (mg/kg) in the bacon after at 0, 7, 15, 22, 30, and 45 days of refrigerated storage.

Biogenic Amines	Storage Time/Days						Pearson’s Correlation Coefficients	
	Day 0	Day 7	Day 15	Day 22	Day 30	Day 45	*r*	*p*
Tryptamine	-	-	-	-	-	-	-	-
Phenylethylamine	-	-	-	-	-	-	-	-
Putrescine	2.81 ± 0.57 ^c,d^	2.39 ± 0.36 ^d^	3.83 ± 0.58 ^b,c^	2.81 ± 0.70 ^c,d^	4.14 ± 0.47 ^b^	11.69 ± 1.86 ^a^	0.827 *	0.042
Cadaverine	5.75 ± 0.53 ^c^	4.23 ± 0.46 ^d^	5.34 ± 0.60 ^c^	7.50 ± 0.93 ^b^	8.76 ± 1.11 ^a^	9.50 ± 0.94 ^a^	0.892 *	0.017
Histamine	1.38 ± 0.31 ^c^	1.12 ± 0.15 ^c^	1.89 ± 0.27 ^b^	1.16 ± 0.17 ^c^	2.17 ± 0.29 ^a,b^	2.38 ± 0.43 ^a^	0.768	0.075
Tyramine	4.02 ± 0.79 ^c^	4.51 ± 0.93 ^c^	4.21 ± 0.53 ^c^	9.94 ± 1.15 ^b^	14.06 ± 1.93 ^a^	15.94 ± 2.48 ^a^	0.940 *	0.005
Spermidine	2.22 ± 0.45 ^a,b^	2.45 ± 0.42 ^a^	2.20 ± 0.32 ^a,b^	2.22 ± 0.30 ^a,b^	2.29 ± 0.31 ^a,b^	1.85 ± 0.15 ^b^	−0.716	0.110
Spermine	6.26 ± 0.98	7.40 ± 0.77	6.23 ± 0.58	7.02 ± 1.05	7.16 ± 1.12	6.93 ± 0.69	0.336	0.515
Total	22.44 ± 1.22 ^d^	22.10 ± 3.90 ^d^	23.70 ± 1.47 ^d^	30.65 ± 2.05 ^c^	38.58 ± 3.93 ^b^	48.29 ± 3.83 ^a^	0.963 **	0.002

Figures in the table are means and standard error. ^a–d^ Means within a row refer to the significant difference at *p* < 0.05 according to Tukey’s multiple range test. Means in the same row with no superscript letters after them or with a common superscript letter following them are not significantly different (*p* < 0.05). - Not detected in sample. * Significant at the 0.05 level; ** Significant at the 0.01 level.

## Supplementary Materials

The Supporting Information is available: Table S1: The mean sensor responses of bacon at 0, 7, 15, 22, 30 and 45 days. Table S2: Loadings of 18 variables (e-nose sensors) on two significant principal components for smoked bacon.

## Abbreviations

VOCs	Volatile organic compounds
BAs	Biogenic amines
SPME	Solid-phase micro extraction
GC-MS	Gas chromatography-mass spectrometry
HPLC	High-performance liquid chromatography
SSOs	Specific spoilage microorganisms
e-nose	Electronic nose
PLSR	Partial least squares regression
LAB	Lactic acid bacteria
PCA	Principal component analysis
ANOVA	Analysis of variance
HTS	High-throughput sequencing
QA	Quality assurance
QC	Quality control
RSD	Relative standard deviation
LOD	Limits of detection
LOQ	Limits of quantification
