# Supplementary material for: Evolution of Volatile Compounds and Spoilage Bacteria in Smoked Bacon during Refrigeration Using an E-Nose and GC-MS Combined with Partial Least Squares Regression
Source: Molecules. 2018 Dec 11;23(12):3286. doi: 10.3390/molecules23123286 (PMC6320767; doi:10.3390/molecules23123286)
Supplement: Supplementary file 1 [file molecules-23-03286-s001.pdf]

Table S1

The mean sensor responses of bacon at 0, 7, 15, 22, 30 and 45 days.

	Storage time /days							
Sensors	day 0	day 7	day 15	day 22	day 30	day 45	r	P
LY2/LG	0.01±0.00^{d}	0.02±0.00^{cd}	0.00±0.00^{d}	0.07±0.05^{bc}	0.10±0.08^{ab}	0.16±0.04^{a}	0.936**	0.006
LY2/G	-0.04±0.00^{a}	-0.05±0.01^{a}	-0.04±0.00^{a}	-0.14±0.06^{b}	-0.18±0.07^{b}	-0.20±0.03^{c}	-0.922**	0.009
LY2/AA	-0.04±0.00^{a}	-0.04±0.01^{a}	-0.04±0.01^{a}	-0.11±0.04^{b}	-0.13±0.05^{bc}	-0.15±0.02^{c}	-0.927**	0.008
LY2/GH	-0.04±0.01^{a}	-0.06±0.01^{a}	-0.05±0.01^{a}	-0.20±0.09^{b}	-0.26±0.11b^{c}	-0.32±0.04^{c}	-0.945**	0.004
LY2/gCTl	-0.03±0.00^{a}	-0.05±0.01^{a}	-0.04±0.01^{a}	-0.16±0.08^{b}	-0.22±0.10b^{c}	-0.28±0.04^{c}	-0.953**	0.003
LY2/gCT	-0.01±0.00^{a}	-0.01±0.00^{a}	-0.01±0.00^{a}	-0.06±0.03^{b}	-0.09±0.03^{bc}	-0.11±0.01^{c}	-0.941**	0.005
T30/1	0.35±0.01^{c}	0.35±0.01^{c}	0.36±0.01^{c}	0.47±0.06^{b}	0.59±0.09^{a}	0.63±0.05^{a}	0.942**	0.005
P10/1	0.42±0.01^{c}	0.43±0.01^{c}	0.44±0.01^{c}	0.57±0.06^{b}	0.74±0.08^{a}	0.77±0.04^{a}	0.937**	0.006
P10/2	0.29±0.01^{b}	0.29±0.01^{b}	0.30±0.01^{b}	0.38±0.04^{a}	0.42±0.04^{a}	0.41±0.05^{a}	0.893*	0.016
P40/1	0.43±0.00^{c}	0.43±0.01^{c}	0.44±0.01^{c}	0.52±0.04^{b}	0.63±0.06^{a}	0.64±0.04^{a}	0.928**	0.008
T70/2	0.30±0.01^{c}	0.32±0.01^{c}	0.34±0.01^{c}	0.45±0.07^{b}	0.63±0.10^{a}	0.67±0.05^{a}	0.948**	0.004
PA/2	0.46±0.01^{c}	0.45±0.01^{c}	0.47±0.01^{c}	0.55±0.05^{b}	0.69±0.09^{a}	0.71±0.06^{a}	0.928**	0.008
P30/1	0.35±0.01^{c}	0.35±0.02^{c}	0.35±0.02^{c}	0.56±0.10^{b}	0.78±0.10^{a}	0.80±0.06^{a}	0.919**	0.010
P40/2	0.30±0.01^{c}	0.30±0.02^{c}	0.30±0.02^{c}	0.47±0.08^{b}	0.71±0.08^{a}	0.76±0.05^{a}	0.927**	0.008
P30/2	0.18±0.03^{c}	0.25±0.04^{c}	0.18±0.03^{c}	0.59±0.12^{b}	0.81±0.09^{a}	0.86±0.04^{a}	0.910**	0.010
T40/2	0.22±0.00^{b}	0.21±0.00^{b}	0.24±0.00^{b}	0.23±0.03^{b}	0.32±0.02^{a}	0.24±0.07^{b}	0.492	0.322
T40/1	0.36±0.00^{b}	0.34±0.00^{b}	0.38±0.00^{ab}	0.36±0.01^{b}	0.42±0.02^{a}	0.33±0.09^{b}	0.006	0.991
TA/2	0.36±0.01^{c}	0.35±0.01^{c}	0.37±0.01^{c}	0.41±0.04^{bc}	0.48±0.05^{a}	0.44±0.08^{ab}	0.822*	0.044

Note: Figures in the table are means and standard error.

$^{a, b, c, d}$ Means within a row refer to the significant difference ($P < 0.05$).

Table S2

Loadings of 18 variables (e-nose sensors) on two significant principal components for smoked bacon.

**Variable**	**PC1**	**PC 2**
P30/1	0.999	
P40/1	0.999	
T30/1	0.999	
P10/1	0.997	
P40/2	0.994	
P30/2	0.993	
T70/2	0.993	
PA/2	0.990	
LY2/GH	-0.987	0.146
LY2/AA	-0.987	0.138
LY2/G	-0.987	0.105
LY2/gCTL	-0.986	0.166
LY2/gCT	-0.985	0.163
P10/2	0.981	
LY2/LG	0.968	-0.246
TA/2	0.953	0.302
T40/1	0.187	0.979
T40/2	0.704	0.704
Proportion of variance	95.8%	2.3%
